# Physicochemical and Sensorial Characterization of Honey Spirits

**DOI:** 10.3390/foods6080058

**Published:** 2017-07-27

**Authors:** Ofélia Anjos, David Frazão, Ilda Caldeira

**Affiliations:** 1 Instituto Politécnico de Castelo Branco, Apartado 119, 6001-909 Castelo Branco, Portugal; 2 Centro de Estudos Florestais, Instituto Superior de Agronomia, Universidade de Lisboa, 1349-017 Lisboa, Portugal; 3 Centro de Biotecnologia de Plantas da Beira Interior, Apartado 119, 6001-909 Castelo Branco, Portugal; davidmfrazao@hotmail.com; 4 Instituto Nacional de Investigação Agrária e Veterinária, Unidade Estratégica de Investigação e Serviços de Tecnologia e Segurança Alimentar, Laboratório de Enologia, Unidade de Investigação de Viticultura e Enologia, Dois Portos, Quinta da Almoínha, 2565-191 Dois Portos, Portugal; ilda.caldeira@iniav.pt; 5 ICAAM— Instituto de Ciências Agrárias e Ambientais Mediterrânicas, Universidade de Évora, Pólo da Mitra, Ap. 94, 7002-554 Évora, Portugal

**Keywords:** honey spirit, honey, physicochemical characterization, sensory analysis, sensory lexicon

## Abstract

Distilled spirits are usually made from fermented sugar-based materials, such as wines or fermented fruits, but other products can be used, namely berries or honey. In this work, an evaluation of honey spirits is done based on its physicochemical and sensory characteristics. Fourteen honey spirit samples of different brands of honey spirit were purchased at the market and from artisan Portuguese producers. Several analytical determinations, namely alcoholic strength, dry matter, density, total acidity, chromatic characteristics, methanol, acetaldehyde, ethyl acetate and higher alcohols were done to characterize all samples. The results pointed out several differences in physicochemical composition of samples. In general, these drinks are characterized by an alcohol strength between 37.4% and 53.0% and a low methanol content, quite null for most samples. Samples with higher ethanol content corresponded to the artisanal samples. Significant differences (*p* < 0.05) were also observed in the volatile composition and chromatic characteristics suggesting different production technologies. A first list of sensory attributes was obtained for this beverage. Therefore, further research must be done in order to characterize this spirit drink, which has gained market value.

## 1. Introduction

Beekeeping is an important economic activity in Europe. However, given the production costs, it is important to find alternatives to outflow the honey production with an increased profit, like mead production [1]. Honey is a food product with a high amount of carbohydrates [2] and a complex range of substances, namely proteins, lipids, vitamins, phenols and flavonoids [3,4]. However, the chemical composition of honey depends on its botanical origin [5,6]. Given its chemical composition, honey has been used not only as food, but also in apitherapy, in cosmetics, as a food ingredient and in beverage preparation. The production of alcoholic drinks such as honey spirit, likewise mead, seems to be one way to increase honey value [1,7]. At present, honey distilled drinks are barely found on the market, but they have a high added value. 

According to European Union (EU) regulation [8] No. 110/2008, the spirit drink sector is important for consumers, producers and the agricultural sector in the European community, thus research should be done to protect consumers and valorise producers in what regards these beverages’ market. The spirit drinks sector has a strong connection with the agricultural sector in the European community since it constitutes a major outflow for agricultural products [8]. This outlet is a result of the quality and reputation these drinks have gained all over the world throughout time [9]. Mead is the main alcoholic drink produced from honey and it has been reported as very rich in nutritional elements required by humans, to have beneficial effects on the digestion and metabolism, and to aid with anemia and chronic gastrointestinal disease treatment [10]. Honey spirit, with an alcoholic content from 7 to 22% (*v*/*v*), is produced through yeast fermentation of honey must, 22 °Brix water diluted honey with some quality (e.g., fruit pulps or juices) and fermentation chemical additives, the mead then being siphoned/racked, matured and bottled [10,11]. The fermentation process is known to be difficult due to the high sugar content and presence of inhibitory compounds in the must, so yeasts must be carefully selected. As a raw material for honey spirit, mead quality is important because of its influence in the final distilled drink. This quality depends on several factors, such as type of honey, water and additive proportions, yeast selection and concentration and fermentation end point [10,12]. 

Honey spirit is obtained by mead distillation and, according to the EU regulation about spirit drinks, only caramel may be added as a means to adapt color and honey as a sweetener to the final honey spirit drink [8]. The resulting drink must have a minimum alcoholic strength of 35% and the organoleptic characteristics derived from honey [8]. Some research has found details about the optimization of mead production, already reporting to its characterization and mainly concerning yeast selection and honey-must formulation [13,14,15]. However, only one study was found that characterizes the volatile components of industrially produced spirits from three unifloral honeys (*Campanilla*, *Citrus* and *Romerillo*) [16]. These authors verified that the most abundant volatile compound classes were saturated alcohols, ethyl esters of saturated fatty acids and terpenes. The saturated alcohols were the dominant class in terms of total amount in all samples of honey spirits, and the major compounds in this group were 3-methylbutan-1-ol and 2-methylbutan-1-ol. The authors identified the most potent odorants and found remarkable differences in the odorant profile of the spirits proceeding from different honeys.

Aroma compounds’ characterization of distilled spirits is important for quality and authenticity. However, sensory analysis is still necessary to describe and evaluate them [9,17,18]. Congeners, such as acetaldehyde, ethyl acetate, methanol and higher alcohols like butan-2-ol, propan-1-ol, 2-methylpropan-1-ol , butan-1-ol, 2-methylbutan-1-ol and 3-methylbutan-1-ol are volatile substances formed mainly during fermentation and also during maturation of spirits, which additionally may be used to provide both qualitative and quantitative information [19].

Because there are few reports regarding honey spirit, we propose, in this work, the first physicochemical and sensory characterization of honey spirits, sampled from the Portuguese market and from artisanal producers. 

## 2. Materials and Methods 

### 2.1. Samples

Different Portuguese honey spirits were gathered from the market and from artisanal producers. In order to compare different markets, two samples were acquired from Poland and from Brazil. The different samples were coded from M1 to M14. Details about the samples are presented in Table 1.

### 2.2. Standards and Chemicals

Ethanol and methanol were purchased from Merck (Darmstadt, Germany). 

GC-FID standards: Ethyl acetate (CAS N° 141-78-6; purity ≥99.8%) was purchased from Riedel-de-Haen (Seelze, Germany), methanol (CAS N° 67-56-1; purity ≥99.9%) was purchased from Merck (Darmstadt, Germany). 2-methylbutan-1-ol (CAS N° 34713-94-5; purity ≥98%) 3-methylbutan-1-ol (CAS N° 123-51-3; purity ≥98.5%), butan-1-ol (CAS N° 71-36-3; purity ≥99.5%), 2-methylpropan-1-ol (CAS N° 78-83-1; purity ≥99.5%), propan-1-ol (CAS N° 71-23-8; purity ≥99.5%), 2-propen-1-ol (CAS N° 107-18-6; purity ≥98%), butan-2-ol (CAS N° 78-92-2; purity ≥99.5%), 4-methylpentan-2-ol (CAS N° 108-11-2; purity ≥98%) and acetaldehyde (CAS N° 75-07-0; purity ≥99.5%) were purchased from Fluka (Buchs, Switzerland).

### 2.3. Analytical Procedures

To characterize the alcoholic drinks, some analytical determinations were done for all the samples: alcoholic strength, density, dry matter, total acidity, fixed acidity, volatile acidity, pH, chromatic characteristics and methanol, acetaldehyde, higher alcohols and ethyl acetate concentration. Two replicates from the same bottle of honey spirits were analyzed and all the analyses were done in duplicate.

Alcohol strength was determined by distillation and electronic densimetry [20], using an electronic densimeter Model 5000 DMA brand Anton Paar (Graz, Austria). The results are presented as volumetric percentage of ethanol in the beverage.

Density, expressed in grams per milliliter, was evaluated by electronic densitometry [20] according to the method recommended for the spirits, using a densimeter Model 5000 DMA brand, Anton Paar (Graz, Austria). 

Dry extract was evaluated by the method suggested by [20] for spirits, which consists in weighing the residue left by evaporation, at 100 °C, of spirits. The results are expressed in g·L^−1^.

Total acidity was evaluated by colorimetric titration [21]. The results are expressed in grams of acetic acid per liter.

Fixed acidity was evaluated by colorimetric titration of the water solution of dry extract [21]. The results are expressed in grams of acetic acid per liter.

Volatile acidity was determined by calculation (total acidity minus fixed acidity) [20]. The results are expressed in grams of acetic acid per liter.

The pH was determined using a potentiometer (micro pH2002, Crison, Barcelona, Spain), according to the method described by [20].

Chromatic characteristics (CIELab) were determined on a Varian Cary 100 Bio spectrophotometer (Palo Alto, CA, USA) and a 10 mm glass cell, by measuring the transmittance of the sample every 10 nm from 380 to 770 nm, with a D65 illuminant. Based on the transmittance values, parameters such as luminosity (L*), saturation (C*) and chromaticity coordinates (a* and b*) were calculated. The coordinate a* takes positive values for reddish colors and negative values for greenish ones, while coordinate b* takes positive values for yellowish colors and negative values for bluish ones [20]. 

The volatile compounds (methanol, acetaldehyde, ethyl acetate and higher alcohols) of each sample were analyzed by gas chromatography-flame ionization detection (GC-FID) according to the validated method [22]. Compound concentrations were determined by direct injection of the distillate, obtained in the alcohol strength determination. Prior to injection, 1 mL of internal standard solution (4-methylpentan-2-ol) was added to 9 mL of each sample (distillate of honey spirit drink).

The GC-FID analysis was carried out using an Focus GC gas chromatograph (ThermoFinnigan, Milan, Italy) ,) equipped with a flame ionization detector-FID (250 °C) and a fused silica capillary column of polyethylene glycol (DB-WAX, JW Scientific, Folsom, CA, USA), 60 m length, 0.32 mm i.d., 0.25 μm film thickness. The carrier gas was hydrogen (3.40 cm^3^·min^−1^). The samples were loaded (~1 µL) on the injector (200 °C) in split mode (split ratio 1:6). The oven temperature program was 35 °C (for 8 min), then increased at 10 °C·min^−1^ to 200 °C and held at this temperature for a further 1 min. The results were expressed in grams per hectolitre of 100% (*v*/*v*) alcohol, which is abbreviated as AE-absolute ethanol (g/hL AE).

### 2.4. Sensory Vocabulary Development 

Ten trained panellists (7 women and 3 men with ages from 24 to 59), with several years of experience in descriptive sensory analysis of wine brandies, did the sensory description of these distillates. The tasting panel was composed by a group of tasters previously selected and trained according to the international standards [23], comprising detection and recognition of tastes and odors and for quantitative descriptive analysis of wine brandies [24]. This panel also had experience in the sensory evaluation of other alcoholic beverages such as grape mark spirits and alcoholic beverages prepared with a maceration of juniper berries [25]. 

The sensory test was done in two sessions, in a sensory room and in balanced orders to eliminate first-order carry-over effects [26]. The samples were presented to the panel as 30 mL in wine tasting glasses [27] at room temperature and under white natural lighting. Water was provided for mouth rinsing between samples.

Tasters were instructed to evaluate each sample, as they do for other alcoholic beverages, in this sequence: (1) color and appearance, (2) aroma evaluation firstly without glass agitation and also after glass agitation, (3) flavor evaluation by taking a sip of the sample and (4) aftertaste evaluation. The tasters were first asked to individually identify the attributes for visual, olfactory and gustatory descriptions of each sample as specifically as possible, then all of the descriptors were compiled into a list. They were also instructed to avoid hedonic descriptors. 

The small number of hedonic attributes generated (e.g., agreeable or good) and non-pertinent attributes (more, little, too much) were eliminated from the initial list. This initial list was provided to each taster for discussion and similar descriptors were combined because they are synonyms, namely fruity and fruit, floral and flowers. After that, the corresponding relative frequency for each attribute was calculated by dividing the number of samples in which the word is used by the number of the whole set of samples. 

### 2.5. Data Analysis

The results of different parameters measured in honey spirits were submitted to a post hoc test in a one-way analysis of variance with different samples (different producers) as fixed factors. Scheffe’s test was applied for mean comparison.

The results were also subjected to the multivariate analysis, namely principal component analysis (PCA), to compare the similarity between samples.

All the calculations were performed using Statistic from Statsoft (vs. 7.09, Statsoft Inc., Tulsa, OK, USA) and Minitab^®^ 17.1.0 (Minitab Inc., State College, PA, USA).

## 3. Results and Discussion

### 3.1. Physicochemical Analysis

Table 2, Table 3 and Table 4 show the results of the physicochemical determinations done on the honey spirit drink samples. 

Honey spirit sample from Poland (M8) had an alcohol strength of 28% *v*/*v* being the only one below the minimum alcoholic strength value (35% *v*/*v*) defined by the EU regulation for honey spirits [8]. Among the other samples, the one from Brazil (M7) and the one with ageing (M12) had the lowest values, respectively, 37 and 38% *v*/*v*, while the others had values between 41 and 53% *v*/*v*. As can be seen in Table 2, the highest density values correspond to samples with lower alcoholic strength, which is probably due to ethanol, the main alcohol that has a lower density than water. Density differences between samples M7 and M12 with near alcohol strength may reflect the difference in dry matter content. M12 is the sample with a distinctively higher value of dry matter content, which, once again, is explained by the wood ageing process. Total acidity and pH values were quite different between samples, which may mean a technological difference, for example in the raw material or product of origin, but it is also known that pH values may vary in the same spirit drink [25]. Although data is not shown, as expected, volatile acidity was the major contributor to the total acidity of the analyzed samples, moreover the aged honey spirit sample M12 being the one with a higher contribution of fixed acidity, which may be explained again by the ageing process. The pH values varied between 2.47 (M6) and 4.49 (M4) and total acidity values varied between 0.11 g of acetic acid/L (M3) and 1.42 g of acetic acid/L (M6).

Through the analysis of color parameters in Table 3, it may be observed that all samples were quite colorless, with luminosity (L*) values of 100% or near. However, M8 and M12 samples presented a darker color, with values of, respectively, 98.5% and 96.9%. M12 darkness is explained by its wood ageing [28]. Saturation (c*) values are almost all near zero, which means that there is weak color saturation or purity, the only exceptions being M8 and M12 with c* values of, respectively, 5.60 and 11.25, which means a more pronounced color, though still a weak saturation. For all samples, h* degree values are near and above 900, which means a color between yellow and green, but M8 and M12 samples have values nearer the 90 degrees, showing a more defined yellow color and more pronounced color, due to c* parameter value. The *Lch* color model showed a very good agreement with the Lab color model because a* coordinate values are negative and small when compared with b* coordinate positive values for all samples, which means a predominant yellowish color with a small contribution of greenish color for all samples. Moreover, a* and b* values are quite small, with the exception of M8 and M12 samples, which have higher values of, respectively, 5.58 and 11.15, for the b* coordinate, meaning a more pronounced yellow color than the other samples. 

Table 4 shows the volatile composition results. As may be observed, methanol content ranged between none (M8) and 67.4 g/hL P.A. (M12), reflecting the low methanol content of honey spirit when compared with other fruit or fruit derived spirits such as grape marc spirits, 355 and 600 g/hL P.A. [29], and traditional Greek arbutus spirit, 88.92 to 1152 g/hL P.A. [30]. These results should reflect the raw material composition. Actually, it was verified that the mead presented low amounts of methanol, which could increase a little when the fermentation happened in presence of pollen [31]. Methanol is toxic to humans, therefore its maximum concentration in the final distillate is fixed at 1000 g/hL P.A. by EU regulation for grape marc spirits and fruit spirits, but not for honey spirits [8]. High methanol concentrations are often found in fruit spirits due to the enzymatic degradation of pectin during fermentation [9], so the low methanol content of honey spirits was expected. 

Higher alcohols are quantitatively the largest group of volatile flavor compounds in distillates and they are positively involved in distillate sensory qualities if they are not present in high concentration [32]. These compounds are produced as metabolites from the degradation of amino acids and are also called “fusel” alcohols because of their malty and burnt flavor [33]. Butan-2-ol and 2-propen-1-ol contents were zero for all samples and therefore the results are not shown. In relation to butan-1-ol content, samples showed a low concentration of the compound, with the exception of two homemade honey spirits, M14 and M3 samples, which had values of 4.8 and 1.9 g/hL P.A, respectively. Propan-1-ol content was found to be between 14.8 and 30.4 g/hL P.A., which are lower values than the ones found for grape marc spirit, 50.2 and 42.5 g/hL P.A. [29], but higher than for Portuguese arbutus spirit, 18.79 and 11.03 g/hL P.A. [32]. Butan-2-ol, 2-propen-1-ol (allyl alcohol) butan-1-ol and propan-1-ol, when in high levels, are associated with microorganisms’ spoilage of raw materials and/or mashes [34,35] and those high levels were not found in any of the samples. Most of the microorganisms present in honey are in inactive forms because of honey hygroscopicity, hyperosmolarity, acidity, peroxide content and antibiotic activity making it a hard environment for their survival [36]. Isoamyl alcohols, 2-methylbutan-1-ol and 3-methylbutan-1-ol together constitute the major portion of higher alcohols in a distillate and their presence reinforces its structure in the mouth. In some spirit drinks, their quality may be evaluated by the quotients isoamyl alcohol/2-methylpropan-1-ol and 2-methylpropan-1-ol/propan-1-ol, which have to be higher than one [37]. Except for the M8 sample, all samples showed the high representation of amyl alcohols in total volatile content and an above one quotient isoamyl alcohol/2-methylpropan-1-ol. As for the 2-methylpropan-1-ol/propan-1-ol quotient, M3 and M14 homemade samples were the only ones showing it below one and, moreover, those samples also showed the highest contents in 1-butanol and isoamyl alcohols. M8 sample from Poland did not show any “fusel” alcohol and methanol content, suggesting that the distillate is not proceeding from a fermented honey. In fact, a few authors refer to remarkable contents of acetaldehyde, ethyl acetate and fusel alcohols in meads, which could be influenced by the type of honey used and fermentation conditions [11,31,38].

Ethyl acetate and acetaldehyde content varied a lot within the samples analyzed. M8 sample from Poland had no content of ethyl acetate and one of the lowest acetaldehyde contents, 10.4 g/hL P.A. Homemade sample M4 had the lowest value for acetaldehyde content, 5.6 g/hL P.A. and the lowest ethyl acetate content after M8 sample, 22.8 g/hL P.A. M14 homemade sample also exhibited very low values for the two compounds, 15.1 g/hL P.A. and 38.1 g/hL P.A. for acetaldehyde and ethyl acetate, respectively. Samples with the highest values were two homemade samples, M6 and M9, from which acetaldehyde contents of 97.0 and 57.2 g/hL P.A. and ethyl acetate contents of 233.0 and 334.5 g/hL P.A. were obtained. When compared with values obtained by Soufleros et al. [30], who analyzed samples of traditional Greek arbutus spirit from local producers, with contents between 104.5 and 1630.0 g/hL P.A. for ethyl acetate and 34.5 and 270.0 g/hL P.A. for acetaldehyde, honey spirits had similar or lower contents of the two compounds. On the other hand, when compared with values obtained by Cortés et al. [29] for Spanish and Italian grape marc spirits from local distilleries in each country, Galicia and Trento, with average contents of 114 and 75 g/hL P.A. respectively, for ethyl acetate and 58.4 and 63.5 g/hL P.A. for acetaldehyde, the honey spirit samples showed more similar results. The two compounds, when in high concentration, are indicative of an aerobic storage during fermentation processes or an incorrect head fraction separation during distillation [29,30]. Ethyl acetate is the main ester in fermented products and their distillates [34] formed by the esterification of acetic acid and ethanol. Acetaldehyde is the most important carbonyl compound of alcoholic fermentation and is formed as an intermediate compound by degradation of pyruvate [9]. The two compounds are the most volatile compounds and that is why they are presented in high levels in head fraction. High concentrations of both compounds are associated with off-flavors.

**Figure 1:**
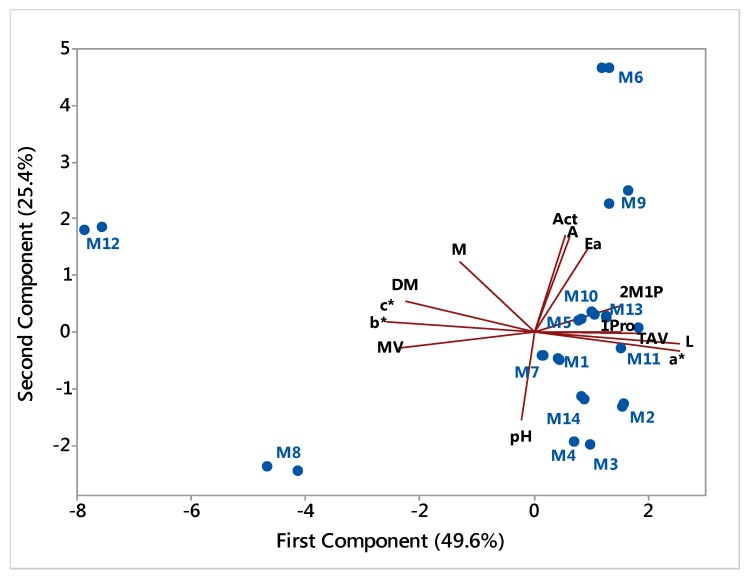
Principal component analysis (PCA) plot with all samples transformed data and interpreting variable vectors. Legend: M: methanol; Act: acetaldehyde; Ea: ethyl acetate; 1Pro: propan-1-ol; 2M1P: 2-methylpropan-1-ol; A: total acidity; TAV: alcohol strength; pH: pH; DM: dry matter; MV: Density; L: luminosity; a* and b*: Lab system coordinates; c*: color saturation.

A PCA of all obtained data, except the one related to 1-butanol content because of its low variability among samples, is plotted in Figure 1 using the two principal components that described better data variation: 49.6% for the first component and 25.4% for the second. In relation to the first principal component, samples M12 and M8 are outlying the cluster formed by the other samples. The variables having more correlation with the first component are the color parameters *L*, a*, b* and c*, the volatile content of propan-1-ol and 2-methylpropan-1-ol and dry matter, alcohol strength and density. As observed before, M12 and M8 showed a more pronounced yellowish color, lower propan-1-ol and 2-methylpropan-1-ol contents and alcohol strength and higher values for density and dry matter. As said before, M12 sample behavior may be explained by its ageing process and M8 sample may not be produced with fermented honey as mentioned on the label, an important fact being that this sample only showed acetaldehyde content among the analyzed volatile compounds. Regarding the second component, M6 and M9 samples were the ones which deviated more from the data cluster. These two samples showed the higher values for acetaldehyde and ethyl acetate content and total acidity and, furthermore, M6 also showed the lowest pH value. These parameters were the ones showing more correlation with the second principal component.

**Figure 2:**
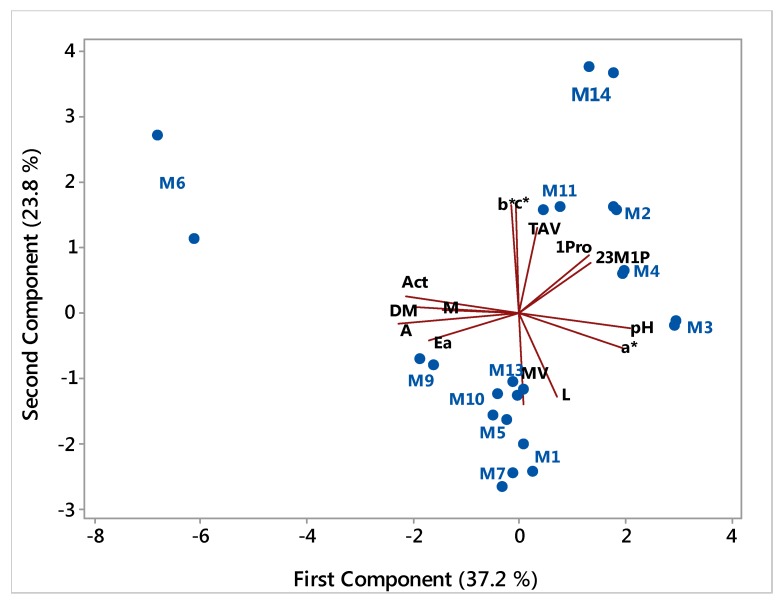
PCA plot with samples transformed data, excluding M8 and M12 samples, and interpreting variable vectors. Legend: M: methanol content; Act: acetaldehyde; Ea: ethyl acetate; 1Pro: propan-1-ol; 2MP: 2-methylpropan-1-ol; 2,3M1B: 2+3-methylbutan-1-ol; A: total acidity; TAV: alcohol strength; pH: pH; DM: dry matter; MV: Density; L: luminosity; a* and b*: Lab system coordinates; c*: color saturation.

A second PCA without samples M12 and M8 was done to eliminate their weight in the overall results and was plotted as shown in Figure 2. In this analysis, the M6 sample continued to stand apart from the other samples. M14 was another sample which distanced itself from the others, apparently because of the high overall fusel alcohol content, low acetaldehyde and ethyl acetate content and probably the color parameters. Two separated clusters may also be observed between the other samples as a result of mainly higher alcohol content, but other variables are likely to be contributing for the separation.

### 3.2. Sensory Vocabulary—Preliminary Results

The sensory analysis done intends to obtain a first approach to the sensory lexicon used for the honey spirits. The lexicon is the group of words that are used to describe the sensory space related to the product [39] and it has been developed for many food products, namely alcoholic beverages, such as wine brandies [40,41]. Some of them are represented and organized in a wheel form such as wine [42], beer [43] and whiskies [44].

By tasting the different honey spirit samples, as explained in materials and methods, a list of 98 attributes were obtained that are presented in Table 5 with the corresponding relative frequency. This initial lexicon comprises 10 attributes related to the visual attributes concerning the color and the appearance of the beverages; 53 attributes regarding the orthonasal aroma and 33 related to the flavor attributes. 

Based on the sensory wheels proposed for other alcoholic beverages [42,43,44], we propose to organize this list (Table 5) in categories considering a first tier, a second tier, to a third tier attribute in some cases (Table 6 and Table 7). These groupings are merely preliminary and further work would need to be conducted to determine the actual relationship of attributes to each other. Concerning the visual attributes, results showed that the majority of these drinks are colorless. Thus, the majority of these spirits appear to only result from a distilling procedure without a wooden ageing process, which is related to many color modifications [17,28], namely the increase of intensity of topaz and a golden color. As for the appearance, the high frequency of the opaline attribute could suggest the absence of stabilization procedures [45], in the production of these honey spirits, which is to be expected once taken into account that several samples proceed from artisanal producers. 

With respect to the orthonasal aroma, the more frequent terms are fruity, floral and sweet associated attributes, meaning that the honey sensory attributes [46,47,48] are highly related with sensory characteristics of the obtained distillates. The two first attributes should be related with odorant compounds pointed out by Pino and Fajardo [16] in honey spirits. In fact, these authors identified in these beverages, as key odorant, several esters, which presented fruity odor notes and terpenic compounds and other esters which exhibited floral notes. The wood related attributes are less frequent in these distillates than in grape brandies that usually go through a maturation process [24,41]. The vegetative/herbaceous attributes are also frequent in these distillates like in other alcoholic beverages, such as whiskies and wine brandies [17,44]. 

Concerning the gustatory attributes, these are focused in the description of retronasal aroma, basic tastes and mouthfeel. The description of retronasal aroma is not as detailed as the orthonasal aroma, but it presents similarities and dissimilarities. In fact, it is remarkable that in the toasted category, the smoky attribute is much more frequent in the retronasal description than in the orthonasal. In Pisco distillates [49], a good correlation was found between the orthonasal and retronasal results with the exception of wood attribute. In wooden aged spirits, the smoky attribute is related to wood derived compounds [24,44]. Given that the majority the samples in this work are not wood matured and that this sensory attribute is used to describe an off-odor in honeys [47], it leads us to hypothesize that these distillates could proceed from used raw material with defective characteristics or that this attribute could result from the distilling process. Further research is needed to understand this matter. 

The basic tastes reported by the panellists are only sweet and bitter in accordance with other distillates [24,44]. With respect to the mouthfeel, the more frequent attributes are burning, smooth and persistency. These attributes have also been used for other distillates [24]. The absence of the astringent is noticeable in the mouthfeel attributes, which could again be related with the absence of a wood ageing process for most of the samples.

These sensory results do not provide the definitive descriptive sensory language for honey spirits but instead intends to be a first approach for future studies. 

## 4. Conclusions

In this study, a first evaluation of commercial and non-commercial Portuguese honey spirit was performed, indicating several physicochemical and sensory profiles on the analyzed samples.

Honey spirits are characterized by an alcohol strength ranging between 37.4% and 53.0% and a methanol content quite null for the majority of the samples, which is an advantage for these beverages.

Given the volatile compositions, the analyzed beverages are characterized by: (i) null concentration of butan-2-ol and 2-propen-1-ol; (ii) low concentration of butan-1-ol; (iii) propan-1-ol content lower than the ones found for grape marc spirit but higher than for Portuguese arbutus spirit; (iv) 2-methylbutan-1-ol and 3-methylbutan-1-ol together constitute the major portion of higher alcohols in the honey spirits analyzed.

In view of the physicochemical analysis, it is possible to conclude that the production of high quality honey spirits is possible. However, it is important to define the best technology/methodology to guarantee quality. Additionally, honey spirit production could constitute an advantage for further uses of wax washing water, which has some pollen and would help in the fermentation process. Since beekeeping production in Portugal has some market bottlenecks, the production of good quality honey spirits might increase producers’ income, given that the product knowledge is enhanced at all levels.

In order to quantify and characterize honey spirit quality, it is important to begin the construction of a lexicon for this beverage. This study presented a list of 98 attributes in a first approach to the sensory lexicon. The most frequent orthonasal attributes related to honey spirits are fruity, floral and sweet, and vegetative/herbaceous. Regarding the gustatory attributes, the most frequent are smoky, sweet and bitter.

This research is yet an early study of the sensory and physicochemical characterization of these beverages that may be helpful in designing future research and for producers to understand the principal quality parameters they need to master in order to better control honey spirit production.

## Figures and Tables

**Table 1:** Sample information.

Code	Type	Provenance	Label Information
M1	commercial	Portugal (Beira Interior—Central region)	Honey spirit; ethanol strength: 40%; product of Portugal
M2	artisanal	Portugal (Algarve—South of Portugal)	
M3	artisanal	Portugal (Central region, mainly produced from honey extracted from residual water of waxes cleaning, mainly *Lavandula* Honey)	-
M4	artisanal	Portugal (North of Portugal)	-
M5	artisanal	Portugal (Beira Interior—Central region)	-
M6	artisanal	Portugal (North of Portugal, mainly from *Castanea* Honey)	-
M7	commercial	Brazil	Honey spirit, ethanol strength: 39%; product in Brazil
M8	commercial	Poland	Honey, based on traditional recipe of distilling honey and herbs; ethanol strength: 38%; produced and bottled in Poland
M9	artisanal	Portugal (Beira Interior—Central region)	-
M10	artisanal	Portugal (North of Portugal)	-
M11	artisanal	Portugal (Beira Interior—Central region)	-
M12	commercial	Portugal	Honey spirit aged in oak wood, ethanol strength: 38%; product of Portugal
M13	artisanal	Portugal (Central region of Portugal, multifloral honey)	-
M14	artisanal	Portugal (Algarve—South of Portugal)	-

**Table 2:** Alcohol strength, total acidity, pH, dry matter and density of the samples analyzed, considering the average and the standard deviation of two determinations for each sample.

Code	Alcohol Strength (% *v*/*v*)	Total Acidity (g of acetic acid/dm^3^)	pH	Dry Matter (g/dm^3^)	Density (g/cm^3^)
M1	41.1 ± 0.01 ^c^	0.51 ± 0.00 ^a^	3.93 ± 0.01 ^d^	0.1 ± 0.01 ^a^	0.946 ± 0.000 ^k^
M2	50.5 ± 0.04 ^j^	0.19 ± 0.01 ^e^	4.23 ± 0.01 ^i^	0.0 ± 0.00 ^a^	0.930 ± 0.000 ^c^
M3	50.2 ± 0.01 ^i^	0.11 ± 0.01 ^b^	4.43 ± 0.01 ^j^	0.0 ± 0.01 ^a^	0.930 ± 0.000 ^e^
M4	53.0 ± 0.00 ^l^	0.26 ± 0.00 ^c^	4.49 ± 0.01 ^k^	0.1 ± 0.01 ^a^	0.945 ± 0.000 ^j^
M5	49.6 ± 0.04 ^g^	0.41 ± 0.01 ^d^	3.91 ± 0.00 ^d^	0.0 ± 0.00 ^a^	0.946 ± 0.007 ^g^
M6	50.1 ± 0.03 ^h,i^	1.42 ± 0.00 ^i^	2.47 ± 0.00 ^a^	0.9 ± 0.01 ^c^	0.930 ± 0.000 ^d^
M7	37.4 ± 0.07 ^a^	0.66 ± 0.01 ^f^	3.62 ± 0.00 ^b^	0.5 ± 0.02 ^b^	0.955 ± 0.000 ^l^
M8	28.0 ± 0.00 ^b^	0.14 ± 0.00 ^a^	4.72 ± 0.00 ^l^	1.4 ± 0.02 ^d^	0.998 ± 0.000 ^n^
M9	46.2 ± 0.00 ^d^	0.91 ± 0.00 ^h^	4.02 ± 0.00 ^f^	0.1 ± 0.01 ^a^	0.938 ± 0.000 ^h^
M10	49.0 ± 0.00 ^f^	0.73 ± 0.00 ^g^	4.18 ± 0.01 ^h^	0.1 ± 0.00 ^a^	0.940 ± 0.000 ^i^
M11	49.8 ± 0.02 ^g,h^	0.48 ± 0.00 ^e^	3.98 ± 0.00 ^e^	0.0 ± 0.01 ^a^	0.930 ± 0.000 ^b^
M12	37.9 ± 0.01^d^	0.40 ± 0.01 ^d^	3.78 ± 0.00 ^c^	24.2 ± 0.12 ^e^	0.986 ± 0.000 ^m^
M13	47.7 ± 0.00 ^e^	0.40 ± 0.01 ^d^	4.46 ± 0.01 ^k^	0.1 ± 0.01 ^a^	0.935 ± 0.000 ^f^
M14	51.2 ± 0.00 ^k^	0.22 ± 0.00^b^	4.12 ± 0.00 ^g^	0.0 ± 0.00 ^a^	0.928 ± 0.000 ^a^

Mean values with the same letter in a row not statistically different.

**Table 3:** Color parameter values of the samples analyzed, considering the average and the standard deviation of two determinations for each sample.

Code	L* (%)	a*	b*	c*	h* (°)
M1	100.0 ± 0.0 ^c^	−0.05 ± 0.01 ^d,e^	0.12 ± 0.06 ^a^	0.13 ± 0.06 ^a^	115.1 ± 5.7 ^a^
M2	100.0 ± 0.0 ^c^	−0.05 ± 0.00 ^d,e^	0.55 ± 0.00 ^a^	0.55 ± 0.00 ^a^	95.6 ± 0.3 ^a^
M3	100.0 ± 0.0 ^c^	−0.01 ± 0.01 ^e^	0.07 ± 0.02 ^a^	0.07 ± 0.02 ^a^	96.2 ± 9.5 ^a^
M4	100.0 ± 0.0 ^c^	−0.05 ± 0.00 ^d,e^	0.48 ± 0.02 ^a^	0.49 ± 0.02 ^a^	95.9 ± 0.6 ^a^
M5	100.0 ± 0.0 ^c^	−0.01 ± 0.03 ^e^	0.10 ± 0.01 ^a^	0.10 ± 0.00 ^a^	98.6 ± 15.2 ^a^
M6	99.9 ± 0.2 ^c^	−0.18 ± 0.02 ^c^	0.53 ± 0.04 ^a^	0.56 ± 0.04 ^a^	108.2 ± 0.9 ^a^
M7	100.0 ± 0.0 ^c^	−0.02 ± 0.02 ^e^	0.24 ± 0.05 ^a^	0.25 ± 0.05 ^a^	96.3 ± 6.2 ^a^
M8	98.5 ± 0.8 ^b^	−0.70 ± 0.02 ^b^	5.58 ± 0.19 ^b^	5.60 ± 0.18 ^b^	97.2 ± 0.4 ^a^
M9	100.0 ± 0.0 ^c^	−0.07 ± 0.03 ^d,e^	0.20 ± 0.02 ^a^	0.22 ± 0.01 ^a^	110.3 ± 9.0 ^a^
M10	100.0 ± 0.0 ^c^	−0.05 ± 0.03 ^e^	0.14 ± 0.03 ^a^	0.15 ± 0.02 ^a^	109.1 ± 16.4 ^a^
M11	100.0 ± 0.0 ^c^	−0.06 ± 0.02 ^d,e^	0.56 ± 0.03 ^a^	0.57 ± 0.03 ^a^	96.5 ± 2.5 ^a^
M12	96.9 ± 0.3 ^a^	−1.45 ± 0.04 ^a^	11.15 ± 0.42 ^c^	11.25 ± 0.41 ^c^	97.4 ± 0.5 ^a^
M13	100.0 ± 0.0 ^c^	−0.06 ± 0.02 ^d,e^	0.08 ± 0.01 ^a^	0.10 ± 0.01 ^a^	126.0 ± 14.5 ^a^
M14	99.9 ± 0.1b ^c^	−0.02 ± 0.02 ^e^	0.77 ± 0.12 ^a^	0.78 ± 0.12 ^a^	91.6 ± 1.8 ^a^

Mean values with the same letter in a row not statistically different.

**Table 4:** Methanol, acetaldehyde, ethyl acetate and higher alcohol concentration of the honey spirit samples analyzed, considering the average and the standard deviation of two determinations for each sample.

Code	MtOH	Ac	EtAc	1P	2M1P	23M1B	1B
M1	9.0 ± 0.4 ^a^	16.0 ± 0.2 ^a,b^	114.1 ± 2.1 ^c^	21.0 ± 0.3 ^c^	37.5 ± 0.6 ^c^	118.8 ± 1.6 ^c^	0.4 ± 0.0 ^b^
M2	3.0 ± 0.1 ^a^	11.9 ± 0.1 ^a,b^	51.8 ± 1.1 ^a,b^	27.7 ± 0.1 ^d,e^	81.7 ± 0.8 ^f^	190.6 ± 1.6 ^f^	0.5 ± 0.0 ^c,d^
M3	1.3 ± 0.2 ^a^	16.8 ± 0.2 ^a,b^	34.6 ± 0.1 ^a^	24.5 ± 0.1 ^c,d^	23.0 ± 0.1 ^h^	377.3 ± 3.2 ^h^	1.9 ± 0.0 ^g^
M4	7.3 ± 0.1 ^a^	5.6 ± 0.2 ^a^	22.8 ± 0.1 ^a^	21.5 ± 0.3 ^c^	36.5 ± 0.4 ^g^	248.8 ± 3.4 ^g^	1.1 ± 0.0 ^f^
M5	27.2 ± 0.0 ^b^	36.5 ± 0.21 ^c^	119.5 ± 1.1 ^c^	15.1 ± 0.1 ^b^	40.8 ± 0.1 ^b^	62.7 ± 0.4 ^b^	0.5 ± 0.0 ^c,d^
M6	30.0 ± 0.0 ^b^	97.0 ± 1.1 ^e^	233.0 ± 1.9 ^e^	14.8 ± 0.1 ^b^	41.7 ± 0.3 ^d^	126.0 ± 0.4 ^d^	1.0 ± 0.0 ^e^
M7	0.5 ± 0.0 ^a^	11.4 ± 0.0 ^a^	80.7 ± 2.3 ^b^	19.2 ± 0.2 ^b,c^	41.0 ± 0.6 ^d^	127.5 ± 1.9 ^d^	0.5 ± 0.0 ^c^
M8	0.0 ± 0.0 ^a^	10.4 ± 0.2 ^a^	0.0 ± 0.0 ^a^	0.0 ± 0.0 ^a^	0.0 ± 0.0 ^a^	0.0 ± 0.0 ^a^	0.0 ± 0.0 ^a^
M9	31.1 ± 7.9 ^b^	57.2 ± 6.3 ^d^	334.5 ± 40.5 ^f^	26.7 ± 1.0 ^d,e^	56.4 ± 0.4 ^e^	163.4 ± 10.7 ^e^	0.0 ± 0.0 ^a^
M10	23.9 ± 1.3 ^b^	22.8 ± 0.1 ^b^	162.3 ± 0.1 ^c,d^	20.8 ± 0.2 ^c^	50.5 ± 0.6 ^d^	136.8 ± 1.5 ^d^	0.0 ± 0.0 ^a^
M11	0.0 ± 0.0 ^a^	31.3 ± 6.8 ^c^	116.0 ± 22.0 ^c^	26.4 ± 3.5 ^d,e^	73.7 ± 3.8 ^e^	170.7 ± 0.6 ^e^	0.0 ± 0.0 ^a^
M12	67.4 ± 1.0 ^c^	26.3 ± 0.9 ^b,c^	67.4 ± 1.0 ^a,b^	17.2 ± 1.0 ^b^	27.0 ± 1.0 ^c^	102.5 ± 1.1 ^c^	0.0 ± 0.0 ^a^
M13	25.7 ± 0.8 ^b^	40.3 ± 0.6 ^c^	191.3 ± 2.4 ^d,e^	25.0 ± 0.3 ^c,d^	59.3 ± 0.5 ^c^	118.9 ± 0.7 ^c^	0.6 ± 0.0 ^d^
M14	22.3 ± 1.2 ^b^	15.1 ± 0.0 ^a,b^	38.1 ± 0.2 ^a^	30.4 ± 0.1 ^e^	23.5 ± 0.1 ^g^	237.6 ± 1.5 ^g^	4.8 ± 0.0 ^h^

Codes: MtOH—Methanol (g/hL P.A.); Ac—Acetaldehyde (g/hL P.A.); EtAc—Ethyl acetate (g/hL P.A.); 1P—propan-1-ol (g/hL P.A.); 2M1P—2-methylpropan-1-ol (g/hL P.A.); 23M1B—2+3-methylbutan-1-ol (g/hL P.A.); 1B—butan-1-ol (g/hL P.A.). Mean values with the same letter in a row not statistically different.

**Table 5:** List of terms generated by the tasting panel. The relative frequency of each term is presented as a number in the brackets.

Visual Attributes	
	Colorless (87.5); yellow green (37.5); yellow-straw (37.5); golden (12.5); topaz (12.5); greenish (12.5); brown (12.5); clear (100); brilliant (87.5); unclear (opaline) (62.5).
**Olfactory Attributes (Orthonasal)**	
	alcohol (100); fruity (100); honey (100); sweet (100); floral (87.5); herbaceous (75); tails (75); menthol (62.5); wood (62.5); burning (50); dried fruits (50); dry fruits (50); rancid (50); rubber (50); arbutus fruit (37.5); baker’s yeast (37.5); bread (37.5); dried figs (37.5); ethyl acetate (37.5); oily (37.5); pear (37.5); smoke (37.5); toasted/burned (37.5); vanilla (37.5); varnish (37.5); caramel (25); citrus (25); coffee (25); dried raisin (25); fresh vegetables (25); heads (25); soap (25); apple (12.5); apricot (12.5); banana (12.5); carob (12.5); chocolate (12.5); cinnamon (12.5); clove (12.5); clove (12.5); cocoa (12.5); Genisteae tree (12.5); grape mark spirit (12.5); hay (12.5); herbs (12.5); jasmine (12.5); juniper tree (12.5); lemon (12.5); orange (12.5); peach (12.5); rock rose (12.5); rosemary (12.5); spices (12.5); sugar cane (12.5); wild flowers (12.5).
**Mouth Sensory Attributes**	
	sweet (100); bitter (50); smoke (100); toasted/burned (100); fruity (87.5); honey (87.5); wood (87.5); vanilla (50);dried fruits (37.5); floral (37.5); rubber (37.5); soap (37.5); caramel (25); grape mark spirit (25); menthol (12.5); algae (12.5); arbutus fruit (12.5); burned flowers (12.5); burned honey (12.5); fig (12.5); heads (12.5); rock rose (12.5); spicy (12.5); burning (100); smooth (100); persistent (100); unctuous (50); body (50); spicy (50); harsh (37.5); fresh (37.5); complexity (25); balance (25).

**Table 6:** Proposal of organization of the terms, generated by the panel to the sensory description of honey spirits, based on the sensory wheels of other alcoholic beverages. Visual and olfactory attributes.

Visual Attributes	Color		Colorless; Yellow Green; Yellow-Straw; Golden; Topaz; Greenish; Brown
	Appearance	Clear	Clear; Brilliant
		Unclear	Opaline
Olfactory Attributes (Orthonasal)	Fruity	berry red	arbutus fruit
		tree fruit	pear; apple; peach; apricot
		tropical fruits	banana
		citrus	lemon; orange
		dried fruits	dried raisin; dried figs
		other	carob
	Floral	floral	jasmine; wild flowers; rock rose; rosemary; carnation
	Herbaceous/vegetative	fresh	fresh vegetables; herbs; *Genisteae* tree; juniper tree
		dry	hay
	Solvent/chemical	pungent	alcohol; menthol
		varnish	ethyl acetate
		other	soap
	Sweet associated	sweet	honey; caramel; chocolate; cocoa; sugar cane
	Woody	woody	wood; vanilla
	Toasted/burned	toasted/burned	smoke; coffee
	Spices	spices	cinnamon; clove
	Nutty/dry fruits	nutty/dry fruits	
	Other	other	burning; baker´s yeast; bread; grape mark spirit
	Off odors	tails	rancid; oily
		heads	
		rubber	

**Table 7:** Proposal of organization of the terms, generated by the panel to the sensory description of honey spirits, based on the sensory wheels of other alcoholic beverages. Mouth sensory attributes.

Basic Tastes	Sweet		
	Bitter		
Olfactory attributes (retronasal)	fruity	red fruit	arbutus fruit
		dried fruits	dried fig
	floral	floral	rock rose
	herbaceous/vegetative	fresh	algae
	solvent chemical	pungent	menthol
		other	soap
	sweet associated	sweet	honey; caramel
	woody	woody	wood; vanilla
	toasted/burned(100)	toasted/burned	smoke; burned honey; burned flowers
	spices	spices	
	off-odor	rubber	
		heads	
	other	other	grape mark spirit
Mouthfeel	burning		
	smooth		
	persistent		
	unctuous		
	body		
	spicy		
	harsh		
	fresh		
	complexity		
	balance		
